# Fetus papyraceous: a rare clinical image

**DOI:** 10.11604/pamj.2023.44.28.38689

**Published:** 2023-01-13

**Authors:** Cherukuri Srinidhi, Shubhada Jajoo

**Affiliations:** 1 Department of Obstetrics and Gynaecology, Jawaharlal Nehru Medical College, Datta Meghe Institute of Medical Sciences, Sawangi, Wardha, Maharashtra, India

**Keywords:** Fetus papyraceous, Intrauterine fetal demise, multiple pregnancy

## Image in medicine

Fetus papyraceous is characterized by the intrauterine fetal demise of a twin occurring early in pregnancy, which is then compressed between membranes and the uterine wall. After a while, the fetus becomes mummified and resembles parchment paper. It is a rare complication with an incidence of 1 in 12,000, mostly among twin pregnancies. Predisposing factors may include: multiple pregnancies, use of assisted reproductive techniques, certain intrauterine conditions like membranous cord insertion. Insufficiency of the placenta or twin-to-twin transfusion syndrome. A 26-year-old unbooked para 2 living 1 with 39 weeks 6 days of gestation with previous vaginal delivery came to a casualty with complaints of lower abdomen pain. Blood investigations and term ultrasound were found to be normal. The patient's vitally stable. On examination, per abdomen, adequate contractions with a cephalic presentation, fetal heart rate of 150 beats/min present. Per vaginal examination, the cervix is fully dilated and effaced, and membranes present with the adequate pelvis. After spontaneous rupture of membranes, delivered a live term baby with a 2.6kg birth weight, the baby cried immediately after birth. On examination of the placenta and membranes, there was an incidental finding of a hard, paper-like flattened structure: a shrunken dead fetus-fetus papyraceous. Differential diagnosis of this condition depends on the time of diagnosis antenatal -vanishing twin syndrome in the first trimester, fetus papyraceous in the second trimester, macerated twin in the third trimester.

**Figure 1:**
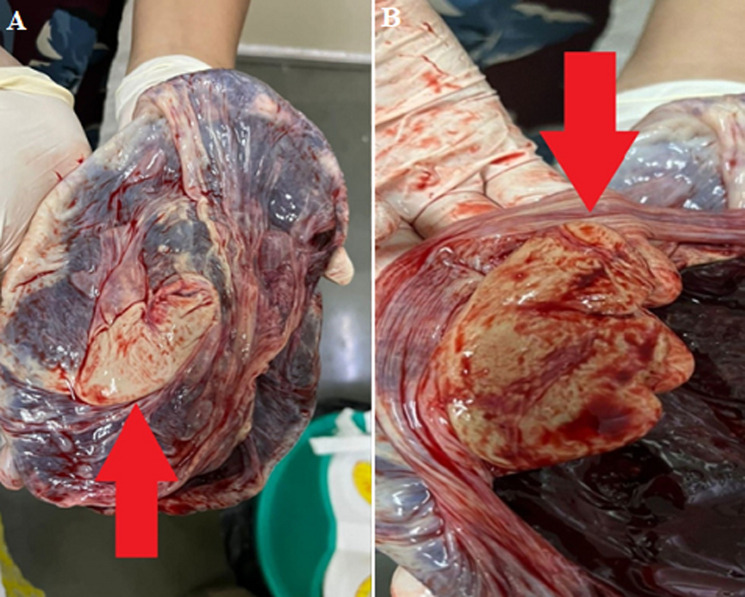
A) fetus papyraceous-hard, B) paper-like flattened structure

